# Editorial: Big Data analytics to advance stroke and cerebrovascular disease: a tool to bridge translational and clinical research

**DOI:** 10.3389/fneur.2023.1347654

**Published:** 2023-12-18

**Authors:** Alexis Nétis Simpkins, Hari Kishan Reddy Indupuru, Sean Isaac Savitz

**Affiliations:** ^1^Department of Neurology, Cedars-Sinai Medical Center, Los Angeles, CA, United States; ^2^Department of Neurology, University of Florida, Gainesville, FL, United States; ^3^Department of Neurology, University of Texas Health Science Center, Houston, TX, United States; ^4^Institute for Stroke and Cerebrovascular Disease, University of Texas Health Science Center, Houston, TX, United States

**Keywords:** Big Data, stroke, machine learning, translational research, intracerebral hemorrhage, stroke risk, ischemic stroke

Big Data analysis has the potential to enhance the high through put processing required to better phenotype patient outcomes post treatment, select potential therapeutic targets, and refine biomarker selection for risk assessment and disease monitoring (1). With data registries, more advanced imaging, data storage tools, and more detailed electronic clinical documentation, robust analysis can be conducted with large datasets with very granular individual patient level data (1–3). Analysis of large datasets requires special considerations to ensure that the significant associations or findings are clinically meaningful and without bias (1).

Use of a Big Data approach can aid in the discovery of pertinent biomarkers for diagnosis and assessment of stroke risk. Wu et al., used regression modeling to determine which factors were associated with patients with brain infarction detected on magnetic resonance imaging (MRI) in a cohort of 1.4 million patients living in China, demonstrating that there were geographic, sex-related, and metabolic disease risk factors for having infarction detected on brain MRI. Efficacy of anticoagulant type was compared by Lee et al., demonstrating a lower risk of stroke and bleeding complications associated with non-oral vitamin K antagonists. Liao et al. conducted a study including over 5 million patients to confirm the increased risk of stroke in association with markers of insulin resistance. Shu et al. demonstrated that altitude has an increased risk of the development of ischemic changes on MRI and an inverse relationship with risk of clinical events of acute stroke. Yang W.-X. et al. studied the efficacy of several machine learning models to predict genetic stroke risk (LASSO, artificial neural network, random forest, and support vector machine - recursive feature elimination model), showing that there are limitations to using these approaches as their models were limited in their accuracy and specificity. Another approach that can be useful are Mendelian randomization models. Ma et al. were able to demonstrate that genetic variants previously demonstrated to be associated with elevated homocysteine levels were not associated with an increased risk of intracranial aneurysm detection by using several Mendelian randomization models. Zhou et al. were able to use random forest models to better predict risk of subarachnoid hemorrhage in patients with middle cerebral artery aneurysms. Combining imaging and clinical variables can improve patient phenotyping. Guo et al. investigated machine learning models as a diagnostic tool to diagnosis stroke by automation. For example, Li Y. et al. demonstrated that CT imaging features and markers of small vessel disease are predictive of the presence of >10 cerebral microbleeds on MRI. More research is needed before Big Data analysis such as artificial intelligence and machine learning can be more ubiquitously applied to clinical care (2–6).

Having practical models that allow for quick assessment of risk for hemorrhagic conversion and risk factors for hemorrhagic conversion have the potential to help with stratifying risk of revascularization therapies such as thrombolysis as there are still risks even after special considerations for eligibility for thrombolysis are made based on clinical factors such as duration of symptoms (7, 8), medications, imaging, and clinical comorbidities within 4.5 h window and in the extended time window per the American Heart Association Guidelines on acute ischemic stroke management (8). Ren et al. used modeling and area under the curve receiver operation characteristic curve analysis to develop a score for predicting risk of hemorrhagic conversion with thrombolysis with an area under the curve value of 0.82. Yang M. et al. created a nomogram that predicts stroke risk with thrombolysis using a combination of imaging, clinical, and blood biomarkers. Risk of ischemic hemorrhagic conversion associated with thrombolysis is further reviewed by Shao et al..

Machine learning can also be used to parse areas of cerebral hypoperfusion and areas of normal cerebral perfusion, which is information that has been useful in thrombectomy clinical trials and was incorporated into clinical guidelines for patient selection for thrombectomy (8) Machine learning has been investigated for its diagnostic utility. Lin X. et al. demonstrated that early patient characteristics available within the first 24 h of hospital admission can be predictive of early outcomes post thrombectomy. They were able to fine tune those predictions using different models such as a the SHapley Additive exPlanations approach (Lin X. et al.). Modeling can also be useful in investigations on posterior circulation infarction such as basilar artery occlusion. Zhao C. et al. confirmed that risk factors such as atrial fibrillation increase risk of recurrent stroke but do not influence basilar artery thrombectomy outcomes. While, Lin S. et al. developed a nomogram to help predict in which patient's with basilar artery occlusion recanalization would be futile. Zeng et al., also looked at futility, but they focused on thrombectomy outcomes in the anterior circulation using a combination of machine learning models combined with the stacking method. Currently, the American Heart Association only endorses volumetric analysis for thrombectomy patients in the extended 24 h window (8). However, several large core endovascular trials have subsequently demonstrated that even patients with large cores may still have some benefit from thrombectomy (9–11). More research is needed to optimize prediction tools for patient selection for thrombolysis and thrombectomy.

Cost of stroke care is projected to be over $90 billion dollars by 2035 (1, 12). Part of those costs are attributed to extra healthcare costs related to stroke associated morbidity (1, 12). Determining who is at risk of medical complications after a stroke and tailoring a post stroke recovery plan could be quite impactful (1). Ji et al. used modeling to develop a risk score to predict the risk of being diagnosed with a deep vein thrombosis in patients that were hospitalized with an intracerebral hemorrhage, and optimized their score using external cohort validation. Comparison of machine learning models can demonstrate which model provides the best sensitivity and specificity to predict the clinical outcome of interest. For example, Zheng et al. compared several machine learning models to determine which model would be most specific and sensitive for predicting which patients admitted with an intracerebral hemorrhage would have a post stroke course complicated by the development of pneumonia, showing that the Gaussian naïve Bayes and logistic regression models both performed well depending on whether the internal or external validation cohorts were used. Feng et al. demonstrated similar proteins were elevated during thrombotic events (acute myocardial infarction and acute ischemic stroke), identifying markers of inflammation. In a study including over 100,000 intracerebral hemorrhage patients, Zhao J. et al. combined regression analysis with causal mediation analysis to determine driving factors behind sex-related outcomes, showing the hemorrhage location and clinical severity were the strongest driving factors of mortality and morbidity. Gu et al. demonstrated that mortality rates are higher in critically ill patients with intracerebral hemorrhage and low calcium levels. Others have used Big Data analytic approaches to study length of stay, healthcare utilization, and healthcare costs (3). Currently, there are no widely accepted models for predicting morbidity and mortality for clinical purposes.

Big Data analysis has been studied to provide prediction models to improve management and coordination of post-acute care. Resource utilization post stroke and needs can vary in patients after hospital discharge, and best practices for managing stroke recovery can change over time (13). Prediction models can be used to determine which patient characteristics are the most associated with likelihood of hospital re-admission within 30 days (Chen Y.-C. et al.), which can used to better allocate resources and services for patients. Chen Y.-C. et al. compared multiple models and assessed the sensitivity and specificity of machine learning models to select the best machine learning model that predicted readmission within 30 days of hospital discharge. Yarfi et al. propose using mixed methods models and qualitative analysis to assess post stroke rehabilitation outcomes. Boutros et al. demonstrated that depression was associated with recurrent stroke and mortality 1 year after stroke. Another model that can be useful in analyzing clinical trial data is Bayesian Network Meta-analysis. Li Z. et al. evaluated several approaches for addressing post stroke cognitive dysfunction, and found that transmagnetic stimulation and acupuncture could be helpful. Chen R. et al. demonstrated that machine learning can be used to differentiate responses to transcranial magnetic stimulation between patient's during the post stroke recovery phase by using unsupervised hierarchical clustering, which could have utility in tracking post stroke recovery. In addition, several studies have used a Big Data approach for assessing quality of life indices (3).

Big Data analytics is a rapidly evolving field and there are important considerations and pauses that should be factored into data interpretation and application. It is important to be aware of biases that may be present in datasets as a result of patient recruitment (1–6). Even within large datasets, there may be unknown missing confounders. It is important to consider validation of results in different datasets (1–6).

## Author contributions

AS: Writing – original draft, Writing – review & editing. HI: Writing – review & editing. SS: Writing – review & editing.
